# The Influence of Environmental Variables on Home Range Size and Use in the Golden Snub-Nosed Monkey (*Rhinopithecus roxellana*) in Tangjiahe National Nature Reserve, China

**DOI:** 10.3390/ani12182338

**Published:** 2022-09-08

**Authors:** Gang Yao, Yuanying Fan, Dayong Li, Vanessa Hull, Limin Shen, Yanhong Li, Jie Hu

**Affiliations:** 1 Institute of Laboratory Animal Science, Guizhou University of Traditional Chinese Medicine, Guiyang 550025, China; 2 Key Laboratory of Southwest China Wildlife Resources Conservation (Ministry of Education), Institute of Rare Animals and Plants, China West Normal University, Nanchong 637009, China; 3 Guang’an Forestry Bureau, Guang’an 638500, China; 4 Department of Wildlife Ecology and Conservation, University of Florida, P.O. Box 110430, Gainesville, FL 32611, USA; 5 Tangjiahe National Nature Reserve, Qingchuan 628109, China

**Keywords:** environmental variables, endangered species, home range, *Rhinopithecus roxellana*, variation partitioning

## Abstract

**Simple Summary:**

The objectives of our study were to determine the seasonal home range size variation, and to determine the fraction of variation in home range use explained by environmental variables. We found that the core home range size of the golden snub-nosed monkey in Tangjiahe National Nature Reserve varied seasonally. The environmental variables in spring and summer explained a large part of home range use intensity, while the environmental variables in autumn and winter explained a moderate fraction and no fraction of home range use intensity, respectively. Water sources, tree density, and dominant trees of Chinese wingnut (*Pterocarya stenoptera*) were the important environmental factors determining home range use.

**Abstract:**

Accurate descriptions of home ranges can provide important information for understanding animal ecology and behavior and contribute to the formulation of conservation strategies. We used the grid cell method and kernel density estimation (KDE) to estimate the home range size of golden snub-nosed monkeys (*Rhinopithecus roxellana*) in Tangjiahe National Nature Reserve. We also used Moran’s eigenvector maps analysis and variation partitioning to test the influence of environmental variables on home range use. The seasonal home range size was 15.4 km^2^ in spring, 11.6 km^2^ in summer, 13.7 km^2^ in autumn, and 15.6 km^2^ in winter, based on the grid cell method. The seasonal core area of 50% KDE was 9.86 km^2^ in spring, 5.58 km^2^ in summer, 7.20 km^2^ in autumn, and 4.23 km^2^ in winter. The environmental variables explained 63.60% of home range use intensity in spring, 72.21% in summer, 26.52% in autumn, and none in winter, and some environmental variables contributed to the spatial variation in home range use intensity. Water sources, tree density, and dominant trees of Chinese wingnut (*Pterocarya stenoptera*) were the important environmental factors determining home range use. These environmental factors require protection to ensure the survival of the golden snub-nosed monkey.

## 1. Introduction

A home range is the total area used by an animal, and encompasses resources that affect an animal’s fitness, including food, potential mates, and safe sites [1,2]. An accurate description of home range is beneficial for the formulation of conservation strategies to protect endangered animals. Some important factors influencing home range size in mammals include season [3,4], food availability [4,5,6,7,8], latitude [9], and human disturbance [10].

Seasons influence home range variation in many primates. Japanese monkeys (*Macaca fuscata fuscata*) have different home range sizes across the four seasons [3]. The home range size of blue-eyed black lemur (*Eulemur flavifrons*) varied significantly between dry and wet seasons [11]. Lion-tailed macaques (*Macaca silenus*) have the largest home range size in summer compared with the other seasons [12].

Food availability is an important environmental variable in ecological studies in birds [13], reptiles [14], and mammals [15,16]. Studies suggested that food availability plays a key role in determining home range size in many primates [17,18,19]. Home ranges are smaller where resources are abundant than where they are limited. Northern pig-tailed macaques (*Macaca leonina*) tend to increase their home range when food is scarce, while they decrease their home range when consuming human food [17]. Japanese monkeys shrink and change their home range size from separate winter and summer ranges to a single, year-round range when supplemental feeding is heavy [20]. The Yunnan snub-nosed monkey (*Rhinopithecus bieti*) has a small home range when fruits, which comprise a major part of the diet, are plentiful and a large home range size when food is scarce [21]. Patchy, high-quality food resources also increase the distance traveled per day in some primates, such as muriquis (*Brachyteles arachnoides*) [22], Yunnan snub-nosed monkeys [23], and Taï chimpanzees (*Pan troglodytes verus*) [24], resulting in a relatively large home range. Primates may travel less on cold days to reduce energy loss when food is scarce [23], and a short travel distance usually means a small home range size [25], resulting in a small home range [2]. Aside from the influence of food resources, hot days are associated with a relatively small home range size for some primates, such as the white-faced capuchin (*Cebus capucinus*) [26,27].

Latitude is another factor contributing to home range size variation [9]. Latitude may act as a surrogate variable for food resource. Higher plant food abundance is observed at lower latitude sites than in higher latitude sites in the northern hemisphere [28], which may lead to a small home range size at low latitudes. For example, in one study, rhesus monkeys (*Macaca mulatta*) at a lower latitude site had a much smaller home range size compared to that of a group at a higher latitude site [29,30].

Anthropogenic activities affect the home range size in some primates. For example, studies showed that the home range size of indri (*Indri indri*) decreased when habitat disturbance increased in Betampona Nature Reserve [31]. Dian’s tarsiers (*Tarsius dianae*) enlarge their home range where anthropogenic effects are increasing [32]. Consequently, studies on animals without anthropogenic disturbance are able to give an accurate description of home range size.

Animals do not use their home ranges evenly. They use some habitat patches more frequently than others. Some environmental variables are important determinants of home range use. For example, water sources influence home range use by primates such as tufted capuchin monkeys (*Cebus paella nigritus*) [33] and red-fronted lemurs (*Eulemur fulvus rufus*) [34]. The forest canopy also influences home range use; for example, samango monkeys (*Cercopithecus mitis erythrarchus*) select habitats with higher canopy density to reduce the risk of predation [35].

**Figure 1:**
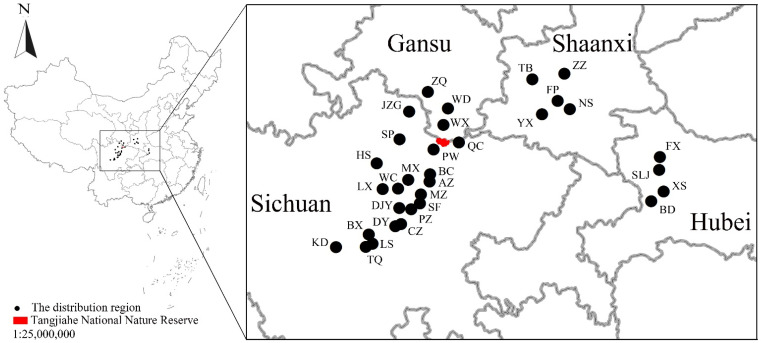
The location of Tangjiahe National Nature Reserve, China. JZG, Jiuzhaigou; SP, Songpan; HS, Heishui; PW, Pingwu; QC, Qingchuan; BC, Beichuan; MX, Maoxian; WC, Wenchuan; LX, Lixian; AZ, Anzhou; MZ, Mianzhu; DY, Dayi; SF, Shifang; DJY, Doujiangyan; PZ, Pengzhou; CZ, Chongzhou; TQ, Tianquan; LS, Lushan; BX, Baoxing; KD, Kangding; WX, Wenxian; ZQ, Zhouqu; WD, Wudou; FP, Foping; YX, Yangxian; ZZ, Zhouzhi; TB, Taibai; NS, Ningshan; SLJ, Shenlongjia; FX, Fangxian; XS, Xingshan; BD, Badong.

The golden snub-nosed monkey (*Rhinopithecus roxellana*) is a semiarboreal, temperate forest-dwelling species endemic to China [36]. The distribution regions for the species include Jiuzhaigou, Songpan, Heishui, Pingwu, Qingchuan, Beichuan, Maoxian, Wenchuan, Lixian, Anzhou, Mianzhu, Dayi, Shifang, Doujiangyan, Pengzhou, Chongzhou, Tianquan, Lushan, Baoxing, and Kangding in Sichuan; Wenxian, Zhouqu, and Wudou in Gansu; Foping, Yangxian, Zhouzhi, Taibai, Ningshan in Shaanxi; and Shenlongjia, Fangxian, Xingshan, and Badong in Hubei (Figure 1) [37,38,39,40]. Three subspecies inhabit three isolated regions: the Moupin golden snub-nosed monkey (*R. roxellana roxellana*) in Sichuan and Gansu; the Hubei golden snub-nosed monkey (*R. roxellana hubeiensis*) in Hubei; and the Qinling golden snub-nosed monkey (*R. roxellana qinlingensis*) in the Qinling mountains, southern Shaanxi [38]. It is an Endangered species [36] that has been prioritized for protection by the Chinese government and is listed in CITES Appendix I (https://cites.org/eng/app/appendices.php (accessed on 6 September 2022)) due to threats of habitat destruction and hunting. It has a seasonal and diverse diet and subsists on over 100 plant species and a variety of plant parts, including buds, young leaves, fruits, flowers, and bark [4,41,42,43,44,45].

Golden snub-nosed monkeys have a larger home range than that of other colobines [46], such as langurs (*Presbytis* spp.) (usually <5 km^2^) [47] and guerezas (*Colobus guereza*) (<10 km^2^) [48], in part because of the large group size but also due to the wide variation in food resources foraged throughout the year [45]. Golden snub-nosed monkeys usually travel much shorter distances for food during seasons when their food resources are abundant than in seasons when food resources are scarce [4], leading to a small home range size [25]. The home range size of the species also varies among groups in different locations [49]. A group of nearly 200 individuals in Baihe Nature Reserve had a home range of 51.42 km^2^ [50]. A group estimated to be made up of 100–120 individuals in Qingmuchuan Nature Reserve occupies a home range of 20.35 km^2^ [44]. A group of >112 individuals in Zhouzhi Nature Reserve has a home range size estimated to be 18.3 km^2^ [51]. Seasonal home range size variation was also observed in golden snub-nosed monkey at a relatively high latitude at the north slope of Qinling mountains, and the group has the largest home range size in spring, the smallest in winter, and moderate in autumn and summer [4].

Many environmental variables influence home range use in golden snub-nosed monkeys, including distance to a water source, the height of large trees, canopy density, and type of forest, while the diameter at breast height (DBH) of large trees also affects habitat use [52,53]. Golden snub-nosed monkeys usually select habitat <300 m from a water source, with large trees with a height of 10–29 m and diameter of 16–30 cm, with a canopy density of 30–80%, and in deciduous broadleaf forest or coniferous–broadleaf mixed forest [52,53]. Moreover, our preliminary observations in Tangjiahe National Nature Reserve, Sichuan, China, suggested that the habitat selected by golden snub-nosed monkeys also included primary forest, large trees, dead trees, fallen logs, tree stumps, and small shrubs. However, the contribution of environmental variables influencing the home range use intensity of golden snub-nosed monkeys is unknown.

We studied the home range size and use in a group of Moupin golden snub-nosed monkeys in Tangjiahe National Nature Reserve, Sichuan, China. Our objectives were: (i) to determine whether the home range size varied with seasonal food resource variation; and (ii) to determine the fraction of variation in home range use explained by environmental variables (water source, large trees, canopy density, type of forest, dead trees, fallen logs, tree stumps, and shrubs) in the habitat selected by golden snub-nosed monkeys.

## 2. Materials and Methods

### 2.1. Study Area

Tangjiahe National Nature Reserve is in Qingchuan, Guangyuan, Sichuan, China (104°37′–104°53′ E, 32°32′–32°41′ N) (Figure 1). The local geography consists of alpine valleys making up the transition zone between the Qinghai–Tibet Plateau and the Sichuan Basin. Tangjiahe is approximately 400 km^2^ in area [54,55] and is adjacent to Baishuijiang National Nature Reserve in Gansu Province to the north and Dongyanggou Nature Reserve in Sichuan Province to the east [56]. The elevation in the reserve ranges 1150–3864 m. Tangjiahe supports evergreen broadleaf forest (elevation: <1600 m), evergreen and deciduous broadleaf mixed forest (elevation: 1600–2000 m), broadleaf–conifer mixed forest (elevation: 2000–2300 m), coniferous forest (elevation: 2300–2500 m) and meadow (elevation >2500 m) [54,55]. The mean annual rainfall ranges 1100–1300 mm [57]. We classified the seasons in the alpine area based on the alpine daily mean temperature, with spring defined by months of April to May, summer by months of June to August, autumn by months of September to October, and winter by months of November to March of the next year [58,59].

### 2.2. Sampling Design

Approximately 14 groups of golden snub-nosed monkeys live in the Tangjiahe National Nature Reserve [60]. Group sizes ranged from 20 to 100 individuals in 2011, and the total number of individuals was 640 [60]. Our previous studies suggested that the largest group increased to 138 individuals in 2014 and inhabited the core area of Tangjiahe National Nature Reserve, with no human disturbance, unlike other groups [58]. We studied this group, which comprised 16 adult males, 48 adult females, 11 subadult females, 3 subadult males, 36 juveniles, and 24 infants [58].

We conducted fieldwork from July 2015 to October 2016. We made preliminary observations of the study group to acclimatize them to our presence from July to October 2015 and observed them from November 2015 to October 2016. We followed the group from dawn to dusk on each sampling day (07:00–19:00) and observed the monkeys at 50–200 m, parallel to their movement [44]. We used a grid size of 200 m × 200 m to track the study group; this grid size is approximately the spread area of the study group [4]. We tracked the grid cells used by overlaying the 200 m × 200 m grid system on a topographic map (1:50,000) [51]. We recorded coordinates using a handheld global positioning system (GPS) receiver (Garmin, GPSMAP60CSX, Shanghai, China) every 30 min. We estimated the central location coordinates of the study group by the compass bearing and distance (detected by a compass and a telemeter) on the topographic map. In addition, we recorded and geolocated any fresh food residues or fresh feces left by the study group and included these sites in the home range; all the site information was ascertained by our actual observation through binoculars before arriving at the observation sites. We used these coordinates of the study group to analyze home range size.

We used ArcGIS 10.3 (ArcGIS Desktop: Release 10.3; Environmental Systems Research Institute: Redlands, CA, USA, https://desktop.arcgis.com/zh-cn/arcmap/10.3/main/get-started/whats-new-in-arcgis.htm (accessed on 6 September 2022)) [61] and Home Range Tools (HRT) version 2.0 [62] software to determine the monkeys’ home range. We imported all recorded location coordinate data into Home Range Tools in ArcGIS 10.3 to model the home range size. We used fixed kernel density estimations (KDE) to measure the home range use distribution [63,64]. We defined the home range size as 95% KDE isopleths and core areas of the home range size as 50% KDE isopleths (contour lines) [65].

We recorded the plant species eaten by the monkeys using instantaneous scan sampling from one side of the study group to the opposite side, at 15 min intervals [66]. We also collected fresh food remains on the ground to identify species. Simultaneously, we recorded the dominant tree species at the foraged place.

We established a set of sampling quadrats (20 m × 20 m) at the location of habitat patches used in the 200 m × 200 m grid cells. We also established control quadrats at the central location of the 200 m × 200 m grid cells, which the study group did not use. We recorded the following parameters for each quadrat: elevation, type of forest, primary forest or secondary forest, number of dead trees, number of fallen logs, number of tree stumps, distance to the nearest water source, tree height, canopy density, mean DBH of trees, tree density, dominant tree species, shrub height and shrub cover (Table 1). We used the sampling points method to measure canopy density [67]. We marked a set of sampling points at an interval of one meter along two vertical diagonal lines in the quadrat. We calculated canopy density using the number of shaded sampling points divided by the total number of sampling points. We used the Braun-Blanquet cover-abundance scale method to measure shrub cover [68].

### 2.3. Data Analysis

We marked group movements on the map and counted the number of times the group used each of the grid cells, considering any cells with a value of one or greater to be within the home range. We added grid cells that had zero values but that were between two locations where we recorded the study group and where there was no other way of passage between the two locations. We calculated annual and seasonal home range sizes by multiplying the number of grid cells by the area of each cell (40,000 m^2^).

We investigated the correlation between the dominant tree species and the foraged tree species to determine whether the foraged tree species were the dominant trees in the foraged habitat patches. The dominant tree species and the foraged tree species are two multistate qualitative descriptors. We binary-coded these two multistate qualitative descriptors as two sets of dummy variables [69] and placed them in two dummy matrices using the function model.matrix in R 3.5.0 software (R Core Team, Vienna, Austria. URL https://www.R-project.org/ (accessed on 6 September 2022)) [70]. We tested the correlation of the two dummy matrices to determine the correlation between the dominant tree species and the foraged tree species using a Mantel test with the mantel.rtest function in R (Monte Carlo test, *n* = 999) [70].

Variation partitioning analysis is used to apportion the variation in response variable sets among two or more explanatory variable sets that may include environmental variables, spatial variables, or temporal variables [69]. The fraction of variation in response variable sets explained by different explanatory variable sets is estimated by the adjusted coefficient of multiple determination (adjusted R^2^). The adjusted R^2^ that provides an unbiased estimate is used to estimate the fraction of variation by partial regression.

The multiscale analysis involves one group method of the spatial eigenfunction analysis. The spatial eigenfunction analysis method includes one special class of general Moran’s eigenvector maps (MEM) analysis that constructs the MEM model as explanatory data. MEM analysis can be combined with variation partitioning analysis to calculate the degree to which the response variable is explained by explanatory variable [69,71].

We constructed an MEM model and combined this with variation partitioning [71] to study the influence of the environmental variables on the frequency with which the study group used each grid cell, and we defined the frequency as home range use intensity. The quadrats established in the grid cells without use were also included in our analysis, and we recognized the frequency as zero. First, we transformed longitude and latitude coordinates into Cartesian coordinates. We defined the home range use intensity as the response variable. We constructed MEM models based on the central coordinates of the grid cells. Calculation of an MEM model includes a connectivity matrix B and an edge weighting matrix A. We used the method provided in previous studies to select the best model [72]. We constructed the connectivity matrix B based on inclusion circles of increasing radii around each point, and the edge weighting matrix A using the complement of the power of the distances, the concave-down function f_2_ [72]. Then, we constructed a spatial weighting matrix W by computing the Hadamard product of the matrix B and matrix A, and a truncated matrix D_trunc_ by replacing the zero values in the matrix W by 4 times the threshold value. The threshold value is the length of the largest edge of a minimum spanning tree that is computed by a matrix D = [1 − s_ij_] in which s_ij_ is the similarity coefficients in concave-down function f_2_ [69]. Finally, we obtained the best MEM model (including several MEM eigenfunctions) by computing the principal coordinates of matrix D_trunc_. The eigenvalues of MEM eigenfunctions were the MEM variables. We used the positive MEM variables as spatial predictors in later variation partitioning analysis. We observed seven positive MEM variables in spring, seven in summer, nine in autumn, and 19 in winter. In addition, we selected significant canonical axis produced by positive MEM variables, and we regressed the canonical axis on the environmental data collected in the set of sampling quadrats by multiple linear regression to assess whether environmental variables affect spatial variation.

We used variation partitioning to separate variation in the home range use intensity (response variables) among explanatory variables, the environmental data from the set of sampling quadrat, and the spatial predictors. We independently forward-selected the explanatory variables before variation partitioning to delete data with significant linear trends. We used permutation tests to test the significance of unique fractions of each explanatory variable alone.

In addition, we regressed the home range use intensity on the environmental data by multiple linear regression to determine which environmental parameters were significantly related to the home range use.

We performed all statistical analyses using R software [70].

## 3. Results

### 3.1. Seasonal Home Range Size Variation

**Figure 2:**
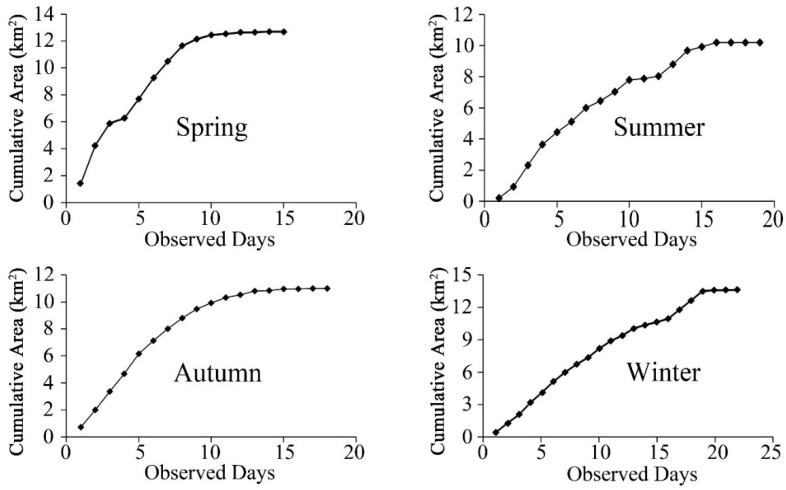
The relationship between cumulative home range area and golden snub-nosed monkey observation days in Tangjiahe National Nature Reserve, China, from November 2015 to October 2016.

Our tracking days were relatively even across seasons (spring: 15 days; summer: 19 days; autumn: 18 days; winter: 22 days; X^2^ = 1.35, *p* = 0.72, df = 3). The cumulative area barely increased in the last five days of observation in all seasons, and there was no significant difference in the accumulated area of the last five observation days in each season (spring: X^2^ = 0.00, *p* = 1.00, df = 4; summer: X^2^ = 0.00, *p* = 1.00, df = 4; autumn: X^2^ = 0.00, *p* = 1.00, df = 4; winter: X^2^ = 0.058, *p* = 1.00, df = 4) (Figure 2). These results collectively suggested that our fieldwork was sufficient to determine the home range in each season.

**Figure 3:**
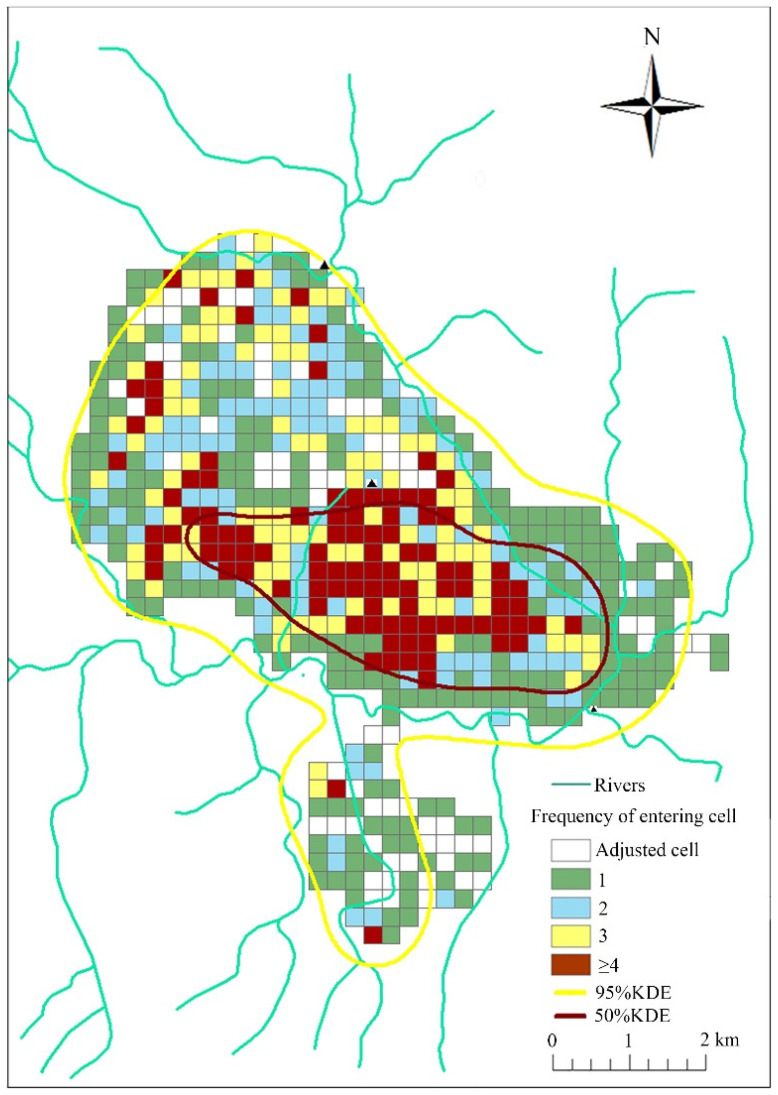
The annual home range size of a golden snub-nosed monkey group (*n* = 138 individuals) in Tangjiahe National Nature Reserve, China, based on the grid cell method and kernel density estimation, November 2015 to October 2016.

**Figure 4:**
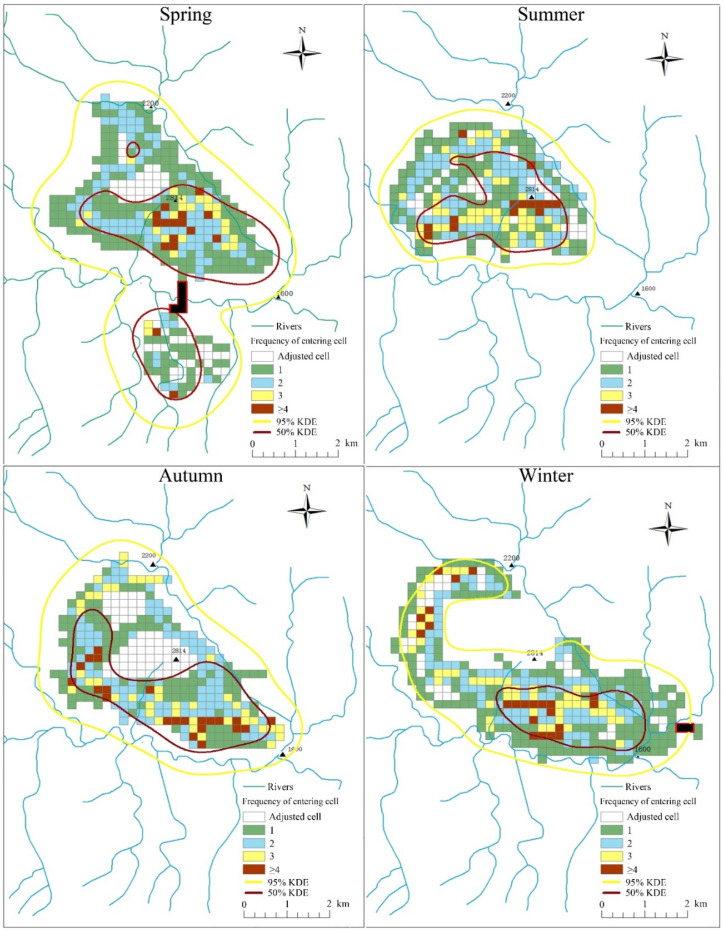
Seasonal home range size of a group of golden snub-nosed monkeys (*n* = 138 individuals) in Tangjiahe National Nature Reserve, China, based on the grid cell method and kernel density estimations for November 2015 to October 2016; adjusted cells included only food residues and feces; black grid cells outlined in red indicate the smallest number of cells between two disparate home range areas with no other way of passage between the two parts (spring and winter).

The golden snub-nosed monkey had a large annual home range (Figure 3). There was significant difference among different seasons in home range size based on 50% KDE isopleths (X^2^ = 10.16, *p* = 0.017, df = 3), but no significant difference based on grid systems (X^2^ = 1.20, *p* = 0.75, df = 3) (Table S1; Figure 4), or the 95% KDE home range (X^2^ = 6.64, *p* = 0.084, df = 3) (Table 2).

### 3.2. Food Species and Food Abundance

The food foraged by the golden snub-nosed monkey varied across the four seasons (Table S2). The study group mainly foraged on bark, buds, and some tender leaves in spring; some bark pieces, many bamboo shoots, tender leaves, and a small portion of fruits in summer; many fruits, some leaves and bark pieces in autumn; and many bark pieces and small buds in winter (Table 3).

The tree species eaten in each season were significantly and strongly positively correlated with the dominant tree species in the habitat patches in spring and winter (spring, r = 0.91, *p* < 0.001, *n* = 64; winter, r = 0.85, *p* < 0.001, *n* = 65), and significantly and moderately positively correlated in summer (r = 0.59, *p* < 0.001, *n* = 39) and autumn (r = 0.61, *p* < 0.001, *n* = 94).

### 3.3. Relationships between Environmental Variables and Home Range Use Intensity

We observed that some of the environmental variables affected spatial predictors. Dominant trees of masson pine (*Pinus massoniana*), primary forest, and elevation may have positive effects on the spatial predictors, and dominant trees of beech (*Fagus longipetiolata*) and basswood (*Tilia tuan*) may have negative effects on the spatial predictors (Table S3). The results suggested that the spatial variation in the home range use intensity may be affected by some environmental variables.

**Figure 5:**
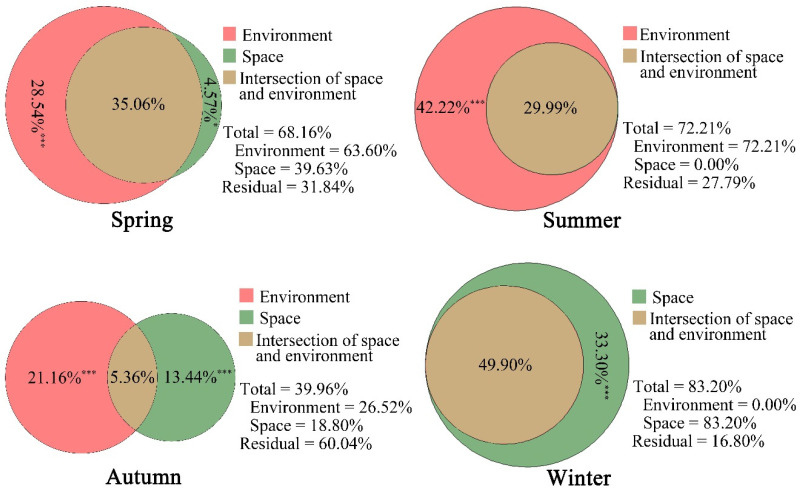
Variation partitioning of home range use intensity by golden snub-nosed monkeys into an environmental component and MEM spatial components (space) in Tangjiahe National Nature Reserve, China, November 2015 to October 2016. *, *p* < 0.05; ***, *p* < 0.001. Values = 0 not shown.

Variation partitioning results suggested that the environmental components explained the home range use intensity more powerfully in spring (63.6%) and summer (72.21%) than in autumn (26.52%) and winter (0.00%) (Figure 5). The explanatory variables of environmental components and space had many intersections to explain the home range use intensity in spring, summer, and winter.

We observed that some environmental variables were related to the home range use intensity (Table 4). The significant environmental variables did not include any foraged food trees, but did include tree density, canopy density, water source, and dominant trees of Chinese wingnut (*Pterocarya stenoptera*).

## 4. Discussion

Our results showed that golden snub-nosed monkeys in Tangjiahe National Nature Reserve had a larger home range size than that found for other colobine monkeys in previous studies (Table 2; Figure 3 and Figure 4) [47]. Home range size may be determined in part by group size. A larger group needs to increase their foraging areas to obtain enough food for all group members [4,21]. Our results are consistent with those of other studies of the same species, with studies recording a large home range size that ranges from 18.3 km^2^ to 51.42 km^2^ [4,44,50,51]. The large home range size observed for the golden snub-nosed monkey is also consistent with those of other snub-nosed monkeys in China. A group of 175 Yunnan snub-nosed monkeys (*Rhinopithecus brelichi*) had a home range size of >20 km^2^ [73], and a group of 125–336 Guizhou snub-nosed monkeys had a home range size of 27.8 km^2^ [49]. These studies suggest that snub-nosed monkeys occupy larger home ranges than other colobine monkeys [46], such as langurs that have ranges from 0.088–0.848 km^2^ [47] and guerezas of 0.053–0.117 km^2^ [48].

Food availability is an important environmental variable influencing home range size of primates [17,18,19]. We found significant differences in home range size calculated from 50% KDE isopleths among the four seasons. This home range variation may be partly attributed to the variation in food availability. In spring, the study group tended to select buds and young leaves as their food, which started to appear in April. Buds and young leaves are sparsely distributed in the home range, requiring the study group to move further distances, resulting in a larger home range size than in other seasons [4]. Abundant food is not a limitation to golden snub-nosed monkeys in summer, and heat stress in summer may restrict the movement [26,27] leading to a smaller home range size than in other seasons. Even if fruits and seeds are abundant in autumn they are usually distributed at different sites, and the golden snub-nosed monkey tends to move long distances to forage on these high quality foods [4], which results in a large home range size in autumn. The golden snub-nosed monkeys mainly foraged on low-quality bark in the winter months when there were no other choices, and they tended to move slowly and over short distances in winter to reduce energy loss [4]. The seasonal home range changes in golden snub-nosed monkeys at lower latitudes were similar to those in other studies at higher latitudes in the Qinling mountains [4].

Regression analysis of the canonical axis produced through MEM variables on environmental variables showed that some environmental variables contributed to the spatial variation in the home range use of the study group. The variation partitioning analysis showed that the environmental components explained a large fraction of the variation in spring (63.60%) and summer (72.21%), a moderate fraction in autumn (26.52%), and none in winter. The variation explained by environmental components was co-explained by the spatial predictors in spring, summer, and autumn, and was explained solely by the spatial predictors in winter. These findings collectively indicated that the environmental components were strongly spatially structured [74], especially the primary forest, elevation, and dominant trees of masson pine, beech, and basswood. The spatial predictors also explained a large fraction of variation, suggesting that the other factors that we did not measure are important drivers of the home range use, which should be determined in more detail in future studies.

We observed that the foraged tree species were positively correlated with the dominant tree species across the four seasons, which suggests that habitat patches selected by the golden snub-nosed monkey are sites potentially including abundant food resources. Interestingly, the foraged tree species were not positively correlated with home range use intensity, but tree density in summer and winter, moderate distance to water source and dominant trees of Chinese wingnut in autumn. Chinese wingnut is a large tree that can provide high tree stratum. Other variables may determine home range use when food is not the factor limiting survival. Studies have observed that ecological variables other than food confine home range use by primates, such as water source availability [33,34] and tree density [53]. Water source availability influences the home range use of golden snub-nosed monkeys, and they regularly drink water from streams in their home range [75]. The golden snub-nosed monkey selects habitats with a high tree density of 10 trees in 20 m^2^ to cross the distance among trees easily [53]. They select patches with large trees to forage leaves, fruits, or bark, and also to use trees over 6 m tall to facilitate fleeing from mammal predators, such as Asiatic golden cat (*Catopuma temmincki*), wolf (*Canis lupus*), red dog (*Cuon alpinus*), and leopard (*Panthera pardus*) [76]. Our results suggest that these environmental variables may have positively effects on the home range use intensity; however, it is worth noting that all *p* values for significant predictors in our model results were significant at the 0.05 level but not the 0.01 level. Consequently, further studies are needed to investigate in detail why the home range use of golden snub-nosed monkeys is not mainly affected by food resources.

## 5. Conclusions

Our study suggested that the core home range size of the golden snub-nosed monkey in Tangjiahe National Nature Reserve varied seasonally. Primary forest, elevation, and dominant trees of masson pine, beech, and basswood contributed to the spatial variation in the home range use of the study group. Water sources, tree density, and dominant trees of Chinese wingnut were the important environmental factors determining the home range use of golden snub-nosed monkeys, and these environmental factors require protection to ensure the survival of the golden snub-nosed monkey.

## Figures and Tables

**Table 1:** Environmental data recorded in quadrats in Tangjiahe National Nature Reserve, China, from November 2015 to October 2016.

Environmental Variable	Description
Elevation (m)	The elevation at the location of the plant species eaten.
Type of forest	Evergreen broadleaf forest, evergreen and deciduous broadleaf mixed forest, deciduous broadleaf forest, coniferous–broadleaf mixed forest, or coniferous forest.
Primary forest or secondary forest	Primary forest: Forest that has never been destroyed by human activities.
	Secondary forest: Forest where large trees were cut down 40 years ago and have since regenerated.
Dead trees	Dead trees in an upright position with DBH ≥ 10 cm.
Fallen trees	Fallen trees with DBH ≥ 10 cm when standing upright.
Tree stumps	Tree stumps with a diameter ≥ 10 cm.
Distance to the nearest water source	0–50 m, 50–100 m, >100 m.
Tree height	Mean height of trees with DBH ≥ 10 cm.
Canopy density	0–25%, 25–50%, 50–75%, and 75–100%.
Mean DBH of trees	Mean DBH of trees with DBH ≥ 10 cm.
Tree density	Stem density of trees with DBH ≥ 10 cm.
Dominant trees species	Tree species with the largest stem density in the quadrat among trees with DBH ≥ 10 cm.
Shrub height	Mean height of shrubs.
Shrub cover	0–25%, 25–50%, 50–75%, and 75–100%.

**Table 2:** Home range sizes of golden snub-nosed monkeys in Tangjiahe National Nature Reserve, China, November 2015 to October 2016.

	No. of Days Observed	No. of Days per Month (Mean ± SE)	No. of GPS Points	Grid Cell Method (km^2^)	50% KDE (km^2^)	95% KDE (km^2^)
Overall	74	6.2 ± 0.8	1763	24.0	6.28	26.95
Spring	15	7.5 ± 0.5	528	15.4	9.86	31.79
Summer	19	6.3 ± 1.5	398	11.6	5.58	15.96
Autumn	18	9.0 ± 2.0	344	13.7	7.20	23.19
Winter	22	4.4 ± 1.2	493	15.6	4.23	19.37

**Table 3:** The ratio of observed diet part on foraged tree species in Tangjiahe National Nature Reserve, China, November 2015 to October 2016.

Seasons		The Ratio of Observed Diet Part (%)				
		Bark	Bud	Tender Leaf	Mature Leaf	Fruit
Spring	April	56.00	24.00	20.00	0.00	0.00
	may	63.63	32.47	3.90	0.00	0.00
Summer	June	69.23	26.92	3.85	0.00	0.00
	July	50.00	15.38	30.77	0.00	3.85
	August	29.41	47.06	5.88	17.65	0.00
Autumn	September	36.58	0.00	0.00	12.20	51.22
	October	36.59	2.44	0.00	20.73	40.24
Winter	November	72.73	0.00	0.00	27.27	0.00
	December	60.00	0.00	0.00	40.00	0.00
	January	75.00	25.00	0.00	0.00	0.00
	February	56.25	43.75	0.00	0.00	0.00
	March	60.53	23.68	10.53	5.26	0.00

**Table 4:** The regression analysis of the home range use intensity on environmental variables in Tangjiahe National Nature Reserve, China, November 2015 to October 2016. The environmental variables with the positive coefficient values suggest a positive influence on the home range use, and the negative coefficient values suggest a negative effect.

Seasons	Significant Environmental Variables	Coefficient	Intercept	t	*p*
Spring	None	None	−19.00	None	None
Summer	Tree density	0.91	−65.70	2.26	0.045
Autumn	Moderate distance to water source (50–100 m)	7.96	29.40	2.12	0.039
	Dominant trees of Chinese wingnut	31.65	29.40	2.14	0.038
Winter	Tree density	0.66	−22.30	2.12	0.043
	Canopy density	−26.02	−22.30	−2.08	0.046

## Data Availability

The data presented in this study are available from the corresponding author upon request.

## Supplementary Materials

The following supporting information can be downloaded at: https://www.mdpi.com/article/10.3390/ani12182338/s1, Table S1: The frequency with which golden snub-nosed monkeys entered 200 m × 200 m grid cells in Tangjiahe National Nature Reserve, China, November 2015 to October 2016; Table S2: Recorded foraged tree species and diet parts of the golden snub-nosed monkey in Tangjiahe National Nature Reserve, China, from November 2015 to October 2016. N, No. of sampling quadrats; Table S3: The regression analysis of canonical axis produced by positive MEM variables on environmental variables in Tangjiahe National Nature Reserve, China, November 2015 to October 2016. The environmental variables with the positive coefficient values suggested a positive influence on the spatial predictors, and the negative coefficient values suggested a negative effect.
