# Supplementary material for: The Influence of Environmental Variables on Home Range Size and Use in the Golden Snub-Nosed Monkey (Rhinopithecus roxellana) in Tangjiahe National Nature Reserve, China
Source: Animals (Basel). 2022 Sep 8;12(18):2338. doi: 10.3390/ani12182338 (PMC9495049; doi:10.3390/ani12182338)
Supplement: Supplementary file 1 [file animals-12-02338-s001.zip › Table S1.pdf]

Table S1. The frequency with which golden snub-nosed monkeys entered 200 m × 200 m grid cells in Tangjiahe National Nature Reserve, China, November 2015 to October 2016.

Seasons	Frequency of entering grid cells			
	Once	Twice	Three times	Four or more times
Spring	183	102	25	16
Summer	108	88	45	14
Autumn	108	103	42	22
Winter	187	97	49	22
Annual	210	113	103	102
