# Supplementary material for: The Influence of Environmental Variables on Home Range Size and Use in the Golden Snub-Nosed Monkey (Rhinopithecus roxellana) in Tangjiahe National Nature Reserve, China
Source: Animals (Basel). 2022 Sep 8;12(18):2338. doi: 10.3390/ani12182338 (PMC9495049; doi:10.3390/ani12182338)
Supplement: Supplementary file 1 [file animals-12-02338-s001.zip › Table S2.pdf]

Table S2: Recorded foraged tree species and diet parts of the golden snub-nosed monkey in Tangjiahe National Nature Reserve, China, from November 2015 to October 2016. N, No. of sam-pling quadrats

| Month (N) | Foraged tree species (frequency) | Diet parts (frequency) |
|---|---|---|
| January (17) | *Salix wallichiana* (5) | Bark (6) Bud (2) |
| | *Acer cappadocicum* (1) | Bark (1) |
| | *Betula utilis* (2) | Bark (2) Bud (1) |
| | *Acer caudatum* (1) | Bark (1) |
| | *Acer laxiflorum* (2) | Bark (2) Bud (1) |
| | *Fagus longipetiolata* (2) | Bark (2) Bud (2) |
| | *Amygdalus persica* (1) | Bark (1) |
| | *Cyclobalanopsis glauca* (2) | Bark (2) |
| | *Litsea chunii* (1) | Bark (1) |
| February (8) | *Betula utilis* (5) | Bark (5) Bud (3) |
| | *Litsea chunii* (2) | Bark (2) Bud (2) |
| | *Rosa* sp. (1) | Bark (1) Bud (1) |
| | *Populus cathayana* (1) | Bark (1) Bud (1) |
| March (27) | *Acer cappadocicum* (5) | Bark (5) Bud (3) Tender leaf (4) |
| | *Picea brachytyla* (1) | Bark (1) |
| | *Betula utilis* (5) | Bark (5) Bud (1) |
| | *Amygdalus persica* (1) | Bark (1) Bud (1) |
| | *Acer franchetii* (2) | Bark (2) Bud (2) |
| | *Populus cathayana* (2) | Bark (2) Bud (1) |
| | *Lindera Limprichtii* (2) | Mature leaf (2) |
| | *Litsea chunii* (1) | Bark (1) Bud (1) |
| | *Ilex fargesii* (1) | Bark (1) |
| | *Fagus longipetiolata* (2) | Bark (2) |
| | *Abies faxoniana* (2) | Bark (2) |
| | Quercus aquifolioides (1) | Bark (1) |

| | | |
|---|---|---|
| April (15) | *Abies faxoniana* (3) | Bark (3) |
| | *Betula utilis* (4) | Bark (4) Bud (4) |
| | *Salix wallichiana* (2) | Bark (2) Bud (2) |
| | *Tilia chinensis* (1) | Bud (1) Tender leaf (1) |
| | *Cyclobalanopsis glauca* (2) | Bud (2) Tender leaf (2) |
| | *Betula albo* (2) | Bud (1) Tender leaf (2) |
| | *Quercus aquifolioides* (1) | Bark (1) |
| May (49) | *Picea brachytyla* (31) | Bark (31) Bud (11) Tender leaf (2) |
| | *Betula utilis* (8) | Bark (8) Bud (6) |
| | *Abies faxoniana* (5) | Bark (5) Bud (3) |
| | *Quercus aliena* (1) | Bark (1) Tender leaf (1) |
| | *Quercus aquifolioides* (1) | Bark (1) Bud (1) |
| | *Salix wallichiana* (1) | Bark (1) Bud (1) |
| | *Quercus aliena* (1) | Bud (1) |
| | *Cerasus duclouxii* (1) | Bark (1) Bud (1) |
| | *Acer oliverianum* (1) | Bark (1) Bud (1) |
| June (6) | *Abies faxoniana* (4) | Bark (4) Bud (3) |
| | *Picea brachytyla* (1) | Bark (1) |
| | *Tilia chinensis* (1) | Bark (31) Bud (11) Tender leaf (2) |
| July (19) | *Picea brachytyla* (2) | Bark (2) |
| | *Abies faxoniana* (4) | Bark (4) Bud (1) |
| | *Litsea chunii* (2) | Fruit (2) |
| | *Salix wallichiana* (3) | Bark (2) Bud (1) Tender leaf (2) |
| | *Tilia chinensis* (5) | Bark (3) Bud (1) Tender leaf (3) Fruit (1) |
| | *Sorbus* sp. (1) | Bark (1) Tender leaf (1) |
| | *Betula albo* (1) | Tender leaf (1) |
| | *Fagus longipetiolata* (1) | Bark (1) Bud (1) Tender leaf (1) |
| August (14) | *Litsea chunii* (6) | Fruit (6) |

| | | |
|---|---|---|
| | *Betula albo* (2) | Bark (1) Tender leaf (1) |
| | *Salix wallichiana*(2) | Bark (2) |
| | *Tilia chinensis* (2) | Mature leaf (2) |
| | *Fagus longipetiolata* (1) | Bark (1) Mature leaf (1) |
| | *Betula utilis* (1) | Bark (1) |
| | *Fargesia denudata* (8) | Bud (8) |
| September (35) | *Quercus aliena* (16) | Bark (4) Mature leaf (2) Fruit (13) |
| | *Fagus longipetiolata* (4) | Bark (2) Mature leaf (1) Fruit (3) |
| | *Picea brachytyla* (1) | Bark (1) Mature leaf (1) |
| | *Tilia chinensis* (1) | Bark (1) Fruit (1) |
| | *Salix wallichiana* (3) | Bark (3) |
| | *Cyclobalanopsis glauca* (4) | Bark (1) Mature leaf (1) Fruit (2) |
| | *Betula utilis* (1) | Bark (2) |
| | *Pinus massoniana* (1) | Bark (1) |
| | *Elaeagnus umbellate* (1) | Fruit (1) |
| | *Quercus aliena* (1) | Fruit (1) |
| October (59) | *Quercus aliena* (14) | Bark (3) Fruit (14) |
| | *Salix wallichiana* (16) | Bark (14) Mature leaf (7) |
| | *Tilia chinensis* (12) | Bark (5) Mature leaf (8) Fruit (9) Bud (1) |
| | *Betula utilis* (1) | Mature leaf (1) Bud (1) |
| | *Betula albo* (1) | Bark (1) |
| | *Sorbus* sp. (3) | Fruit (3) |
| | *Betula albo-sinensis* (1) | Mature leaf (1) |
| | *Acer caudatum* (1) | Bark (1) |
| | *Abies faxoniana* (1) | Bark (1) |
| | *Elaeagnus umbellate* (3) | Fruit (3) |
| | *Litsea chunii* (2) | Bark (2) Fruit (2) |
| | *Cyclobalanopsis glauca* (2) | Fruit (2) |

| | | |
|---|---|---|
| | *Salix eriostachya* (1) | Bark (1) |
| | *Populus cathayana* (1) | Bark (1) |
| | *Cerasus szechuanica* (1) | Bark (1) |
| November (8) | *Salix wallichiana* (6) | Bark (6) Mature leaf (1) |
| | *Tilia chinensis* (1) | Bark (1) Mature leaf (1) |
| | *Betula utilis* (1) | Bark (1) Mature leaf (1) |
| December (5) | *Populus cathayana* (2) | Mature leaf (2) |
| | *Lonicera ligustrina* (1) | Bark (1) |
| | *Rosa* sp. (1) | Bark (1) |
| | *Salix wallichiana*(1) | Bark (1) |
