# Supplementary material for: The Influence of Environmental Variables on Home Range Size and Use in the Golden Snub-Nosed Monkey (Rhinopithecus roxellana) in Tangjiahe National Nature Reserve, China
Source: Animals (Basel). 2022 Sep 8;12(18):2338. doi: 10.3390/ani12182338 (PMC9495049; doi:10.3390/ani12182338)
Supplement: Supplementary file 1 [file animals-12-02338-s001.zip › Table S3.pdf]

Table S3: The regression analysis of canonical axis produced by positive MEM variables on environmental variables in Tangjiahe National Nature Reserve, China, November 2015 to October 2016. The environmental variables with the positive coefficient values suggested a positive influence on the spatial predictors, and the negative coefficient values suggested a negative effect.

Seasons	Environmental variables	Coefficient	Intercept	t	*P*
Spring	Dominant trees of *Pinus massoniana*	0.55	0.15	3.02	0.007
Summer	Primary forest	0.34	-0.59	2.59	0.025
	Dominant trees of *Fagus longipetiolata*	-0.29	-0.59	-3.64	0.004
Autumn	Altitude	0.0003	-0.59	2.07	0.043
Winter	Dominant trees of *Tilia tuan*	-0.73	0.44	-4.85	< 0.001
